# Perception of Changing Habits among Italian Children and Adolescents during COVID-19 Quarantine: An Epidemiological Study

**DOI:** 10.3390/children9060806

**Published:** 2022-05-30

**Authors:** Giulia Bassi, Elisa Mancinelli, Bianca Boldrini, Giada Mondini, Emilia Ferruzza, Daniela Di Riso, Silvia Salcuni

**Affiliations:** 1 Department of Developmental Psychology and Socialization, University of Padova, 35121 Padova, Italy; elisa.mancinelli@phd.unipd.it (E.M.); bianca.boldrini@studenti.unipd.it (B.B.); emilia.ferruzza@unipd.it (E.F.); daniela.diriso@unipd.it (D.D.R.); silvia.salcuni@unipd.it (S.S.); 2 Digital Health Lab, Centre for Digital Health and Wellbeing, Fondazione Bruno Kessler, 38123 Trento, Italy; 3 The Italian Sexology Center (Centro Italiano di Sessuologia, CIS), 40138 Bologna, Italy; jadeworldier@gmail.com

**Keywords:** emotions, social network sites, children, adolescents, COVID-19, quarantine

## Abstract

We used an epidemiological study to explore the perception of change in several psychosocial dimensions during the COVID-19 quarantine. We focused on emotions, use of social network sites (SNSs), family life, important relationships, body functions, and school life. Using snowball recruitment, *N* = 1047 Italian children, pre-adolescents, and adolescents (M = 13.74 ± 3.59) were selected to complete ad hoc online questionnaires. A differential semantic inventory plot was prepared to analyze the emotional experience of children, pre-adolescents, and adolescents during quarantine compared to the pre-quarantine period. The Kruskal–Wallis test was run to assess gender and age differences in emotions experienced, habitual SNS use, and the experience of attending classes remotely. A post hoc Wilcoxon test was performed to compare such differences. Results showed that most of the sample (93.1%) attended classes using technological devices and reported missing their classmates very much (59.3%). Adolescents experienced more negative emotions (M = 3.69 ± 1.33) than pre-adolescents (M = 4.64 ± 1.32), who experienced negative emotions more than children (M = 5.11 ± 1.24). Females were more prone to experience negative emotions compared to males. Adolescents were also the most prolific SNS users (78.1%), particularly female adolescents. Overall, these finding highlight the necessity of preserving the emotional state and relational well-being of youth in these developmental phases by considering their school and social lives.

## 1. Introduction

On 11th March 2020, the World Health Organization declared COVID-19 a worldwide pandemic [1]. In this context, Italy was the second country—after China—to be widely impacted by a COVID-19 outbreak, recording a death rate of 53.5 per thousand population [2]. Of the 230,000 Italians who contracted the virus [1], 2.1% were under the age of 18 [3]. On 10th March 2020, the Italian government expanded the quarantine to cover all of Italy, making Italy one of the first countries worldwide to impose a national lockdown. The sudden restrictions (i.e., massive home confinement; safety measures, such as face masks; and continuous use of disinfectants) forced millions of Italian children, pre-adolescents, and adolescents to stay at home, without going to school or attending any type of outdoor activities, thereby causing significant changes in their psychosocial environment, in particular in terms of social life and interpersonal relationships. For instance, as regards school life, some authors highlighted that based on parent reports, among the different affective states experienced during the lockdown, the highest percentage of children, pre-adolescents, and adolescents showed difficulties in concentrating, followed by feelings of boredom, irritability, nervousness, and restlessness [4,5]. Studies have further found that younger children were more negatively affected by distance learning than older children [6]. In addition, the absence of in-person contacts with their peers, classmates, and even teachers could have a significant impact on children, pre-adolescents, and adolescents’ psychosocial well-being [7,8]. Previous studies showed that the prolonged home confinement affected children, pre-adolescents, and adolescents in terms of symptoms of depression, anxiety, and stress, as well as feelings of worry, anger, fear, fatigue, loneliness, and uneasiness [9,10,11]; these were among the most commonly reported psychological consequences. As such, one can assume that the pandemic might lead to long-term consequences for children, pre-adolescents, and adolescents [12]. The imposed home confinement has been detrimental to children, pre-adolescents, and adolescents’ physical health patterns, in addition to leading to unhealthy sleep routines, sleep disturbances, physical complaints [4,13], and scarce outdoor physical activity [4,8,13]. As such, during the lockdown, technological devices and social network sites (SNSs) were the sole means of social interaction. Consequently, there has been a sharp increase in the use of digital devices, including computers, tablets, and smartphones, by children, pre-adolescents, and adolescents [14], which are necessary for attending distance learning lessons. However, this means that children, pre-adolescents, and adolescents might have been particularly exposed to a potentially problematic overuse of such technological devices [4,14].

With consideration of the above factors, to the best of our knowledge, this study is the first to attempt to comprehensively identify the critical aspects consequent to the quarantine experience, laying the foundations for subsequent interventions. Indeed, the above research findings suggest the potential for significant long-term consequences of the pandemic, particularly those related to social restriction, which is crucial for the healthy psychosocial development of all the age groups considered [7,8], albeit with some differences between groups. Accordingly, the aim of the present study is to explore and assess, both jointly and separately, the psychosocial impact of the quarantine period on Italian children, pre-adolescents, and adolescents, as well as their perception of change related to several psychosocial dimensions, namely family life, important relationships, body functions, and school life. With this study, we further focus on emotions and the use of technological devices and SNSs by considering gender and age differences.

## 2. Materials and Methods

### 2.1. Participants

The present study benefits from a high response rate of participants, ultimately including *N* = 1047 Italian children, pre-adolescents, and adolescents (*n* = 626, 59.8% females) with age ranging between 8 and 19 years (M_age_ = 13.74; SD = 3.59). In particular, *n* = 265 children aged from 8 to 10 years (M_age_ = 9; SD = 0.88), *n* = 293 pre-adolescents aged from 11 to 14 years (M_age_ = 12.43; SD = 1.07), and *n* = 489 adolescents aged between 15 and 19 years (M_age_ = 17.09; SD = 1.42) participated. No participant was excluded.

The three age groups were defined in line with the developmental phases as follows: 8–10 years: children; 11–14 years: pre-pubescents (pre-adolescents) [15]; and 15–19 years: pubescents (adolescents). Nonetheless, it should be noted that children were included from age 8 because at this age, they are expected to be capable of reading autonomously, making them more capable of understanding questions and self-reporting their responses to the questionnaire queries. Instead, the adolescents’ age range (i.e., 15–19) is supported by the WHO [16] definition of adolescents (i.e., from age 15 to 19). Explicit inclusion criteria are reported in the Procedure section below.

### 2.2. Procedure

Two ad hoc surveys were developed and administered between April and May 2020, i.e., concomitant with the COVID-19 quarantine period, through the Google Forms online platform. The ad hoc questionnaires were developed by the senior study authors (S.S., D.D.R., E.F. and G.M.), who are expert psychologists and psychotherapists in the field of developmental psychology. Accordingly, questions were designed through discussion guided by our research, clinical knowledge, and expertise to investigate children’s, pre-adolescents’, and adolescents’ experience of the lockdown, as well as the impact of pandemic-related changes. Both questionnaires also included a differential semantic (DS) inventory [17].

The first questionnaire was aimed at children and pre-adolescents (*“How am I living the quarantine?”*). To complete the questionnaire, participants had to meet the following inclusion criteria: (i) 8 to 14 years of age and (ii) currently living in Italy.

The second questionnaire was aimed at adolescents (*“Adolescents in QUARANTINE!”*). Inclusion criteria were as follows: (i) 15 to 19 years of age and (ii) currently living in Italy.

For either survey, no further inclusion or exclusion criteria were imposed.

The two separate questionnaires were developed in line with differences in the psychosocial functioning of adolescents compared to children and pre-adolescents [18,19]. Accordingly, adolescents (i.e., 15–19 years) received a separate questionnaire from that of children and pre-adolescents, with slight differences in the wording of sentences and graphical design included to better tailor the survey to the population and favor engagement. Moreover, parents of children and pre-adolescents were able to assist them while completing the questionnaire. At the end of the latter questionnaire, a question was administered regarding *“Who filled in the survey?”*. In contrast, adolescents were advised to fill in the questionnaire autonomously to ensure more thoughtful and reliable responses.

Both questionnaires were distributed through a link sent via e-mail, WhatsApp, or SNSs (i.e., Facebook and Instagram), using snowball recruitment among parents, friends, and colleagues through e-mail or smartphones. Before children or adolescents filled in the questionnaires, one parents had to read and check the informed consent, except for adolescents aged 18 years or older, who could give informed consent independently, having reached adult age.

When accessing the questionnaire link, no identifiable data were collected (e.g., name and surname, e-mail address, IP location), thereby ensuring the full anonymity of respondents.

Parents and participants were informed of the study’s general aim and of the anonymity of data collection, which permitted them to withdraw from the study at any moment without any consequence, ensuring the non-use of their data. Data were treated anonymously and analyzed in aggregated form. Both surveys complied with the Declaration of Helsinki (Italian law 196/2003) [20], and data were protected under UE GDPR 679/2016 [21]. The format of the protocol, as well as the informed consent, followed the indication and format suggested by the ethical interdepartmental committee of Padova University for anonymous online research.

### 2.3. Measures

*Child and Pre-adolescent Questionnaire*—*“How am I living the quarantine?”*. The aim of this ad hoc questionnaire was to assess the perception of how habits and emotions had changed during the COVID-19 lockdown in children and pre-adolescents aged between 8 and 14 years and living in Italy.

The questionnaire was administered from 11th April to 20th April 2020.

The first part of the questionnaire was dedicated to demographic information (i.e., gender, age, school, region, family situation, whether they have a pet, hobbies, and sports practice). The second part focused on the perceived differences during quarantine compared to pre-quarantine in several areas of functioning. The investigated areas and sample items were as follows: family life (e.g., *“Compared to before, do you get along with your parents?”*), body functions (e.g., *“Compared to before, are you hungry?”*), important relationships (e.g., *“Compared to before, can you spend time (even via technology) with your friends?”*), school life (e.g., *“Do you miss your classmates?”*), and the use of technological devices (e.g., *“Since you have been home due to COVID-19, the time you spend on Social Networks is…?”*). The questionnaire answer options were tailored to the questions. Answers were either dichotomous (0 = “No”; 1 = “Yes”), referred to nominal scales (e.g., *“Do you have any pets?”*), were multiple-choice (e.g., 1 = “less than before”; 2 = “the same as before”; 3 = “more than before”), or referred to ordinal scales (e.g., *“Do you miss your classmates?”*).

*Adolescent Questionnaire*—*“Adolescents in QUARANTINE!”*. The aim of this ad hoc questionnaire was to assess the perception of how habits, emotions, and well-being changed during the COVID-19 lockdown in adolescents aged from 15 to 19 years and living in Italy.

The questionnaire was administered from 27th April to 5th May 2020.

This second questionnaire evolved from the first one, comprising, as previously reported, the same questions, which were adapted to the relevant age range. The questionnaire answer options were tailored to the questions, as per the children and pre-adolescent questionnaire. Answers were either dichotomous (0 = “No”; 1 = “Yes”), referred to nominal scales, were multiple-choice, or referred to ordinal scales (e.g., 1 = “less than before”; 2 = “the same as before”; 3 = “more than before”).

The included DS in both questionnaires evaluated how participants felt during the quarantine period (e.g., *“Compared to before the COVID-19 isolation, how do you feel now?”*). The DS was adapted to meet the needs of the research issue [17] and following the structure of the *“My Child”* scale [22,23]. The current DS comprises 28 pairs of opposite adjectives clustered into 4 factors (i.e., emotions, activity, satisfaction, and personality) based on a 7-point scale (1, “very much”; 2, “somewhat”; 3, “a little”; 4, “neither one nor the other”; 5, “a little”; 6 “somewhat”; 7 “very much”). Samples of items are as follows: *“Sad–Happy”* or *“Negative–Positive”* (emotions), *“Tired–Energetic”* or *“Inactive–Busy”* (activity), *“Unsatisfied–Satisfied”* or *“Dirty–Clean”* (satisfaction), and *“Rigid–Flexible”* or *“Hated–Loved”* (personality).

Two final questions were asked to the participants, with answers rated on a 10-point Likert scale: *“How badly do you want to go back to your old life on a scale of 1 (not at all) to 10 (very much)?”* and *“How much did you like filling in the questionnaire from 1 (not at all) to 10 (very much)?”*.

### 2.4. Data Analysis

Statistical analyses were performed using R [24] and SPSS Statistics, version 24.0 [25].

The Shapiro–Wilk test was performed to evaluate the normal distribution of variables. Bartlett’s test was performed to evaluate homoscedasticity among the sample subgroups (i.e., children, pre-adolescents, and adolescents).

The main descriptive statistics (i.e., mean, standard deviation, and frequencies) were calculated according to the psychosocial dimensions considered within the overall sample (i.e., family life, important relationships, body functions, school life, use of digital devices and SNSs, and emotions).

Furthermore, the DS inventory plot, as well as the four-factor means (i.e., emotions, activity, satisfaction, and personality), were developed to identify how children, pre-adolescents, and adolescents felt during quarantine in comparison with the pre-quarantine period.

The Kruskal–Wallis non-parametric test was carried out to evaluate gender and age differences with respect to the four factors of the DS questionnaire (i.e., emotions, activity, satisfaction, and personality), the habitual use of SNSs, specific SNSs (i.e., TikTok, Instagram, Facebook, and Twitter), surfing the Internet (e.g., videos or tutorials), the habitual use of videogames, time spent on the computer and/or watching TV during quarantine, and the experience of attending school lessons remotely via digital devices while staying home. A post hoc Wilcoxon non-parametric test was performed to compare and understand the differences between males and females, as well as between the age groups (i.e., children, pre-adolescents, and adolescents).

## 3. Results

### 3.1. Descriptive Analyses

Overall, *n* = 281 (26.84%) participants attended primary school, *n* = 296 (28.27%) attended middle school, *n* = 447 (42.69%) attended high school, and *n* = 4 (0.38%) were in their first year of university. The remaining *n* = 19 (1.82%) were working adolescents. As regards the geographical distribution of the sample, participants from all regions of Italy took part in the current study, of which *n* = 135 (12.9%) live in the northwestern areas of Italy, whereas *n* = 347 (33.1%) in the northeast. Moreover, *n* = 368 (35.1%) live in the central regions of Italy, and *n* = 197 (18.8%) live in the southern and insular regions. Referring to their situation at home, *n* = 432 (41.3%) live in an apartment, *n* = 249 (23.8%) in multi-family houses or in a villa, and *n* = 361 (34.5%) in single-family houses. Most of the sample group has a balcony or a garden (*n* = 978; 93.4%), and *n* = 595 (56.8%) own a pet. Most have at least one sibling and live with their family (*n* = 1027; 98.1%), except for a small number of adolescents (n = 20; 1.9%), who live alone. As regards leisure activities, *n* = 840 (80.1%) usually practice sports, and *n* = 967 (92.4%) report having hobbies.

Among the children and pre-adolescent groups (*n* = 558), 41% (*n* = 432) completed the questionnaire autonomously, whereas 12% (*n* = 126) of parents answered the questions proposed on behalf of their children.

The overall sample (*N* = 1047) reports a mean response of 8.78 (SD = 1.852) to the question *“How badly do you want to go back to your old life on a scale of 1 (not at all) to 10 (very much)?”* and a mean of 7.99 (SD = 1.998) to the question *“How much did you like filling in the questionnaire from 1 (not at all) to 10 (very much)?”*.

### 3.2. Family Life, Important Relationships, Body Functions, and School Life

**Figure 1:**
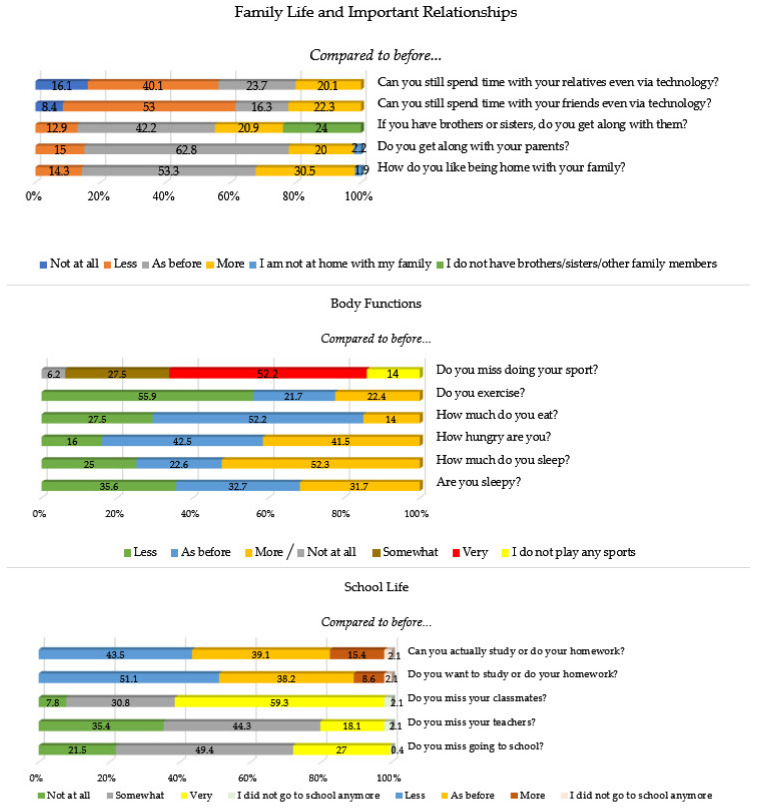
Frequency (%) of the investigated variables related to family life, important relationships, body functions, and school life (*N* = 1047).

Figure 1 shows the overall responses to the questions related to family life, important relationships, body functions, and school life among children, pre-adolescents, and adolescents. Responses related to the pleasure of being at home with parents and siblings during the quarantine show an even and comparable frequency distribution, whereas a higher percentage of the sample reported more pleasure related to staying at home with their family. In contrast, the overall sample reported reduced contact, even via technology, with friends, as well as with relatives (i.e., uncles, aunts, cousins, grandfathers, grandmothers, etc.) outside their immediate family nucleus compared to before the quarantine. As regards body functions, such as sleeping, sleepiness, hunger, food consumption, and lack of sport practices, compared to before the quarantine period, the majority missed their sport activities, reporting less exercise than before the quarantine. Most of the sample (975, 93.1%) attended school lessons using technological devices; the frequency distribution related to the pleasure of attending school using technology is spread across the various response options. Additionally, when it comes to the question regarding whether they miss going to school or their teachers, the frequency distribution is evenly spread across all response items; the sole exception was the question related to their classmates, for which the entire sample reported missing them very much. Finally, the overall sample reported being less inclined to study and less able to effectively do their homework compared to pre-quarantine.

### 3.3. Social Network Site Use before the Quarantine Period versus during the Quarantine Period

**Figure 2:**
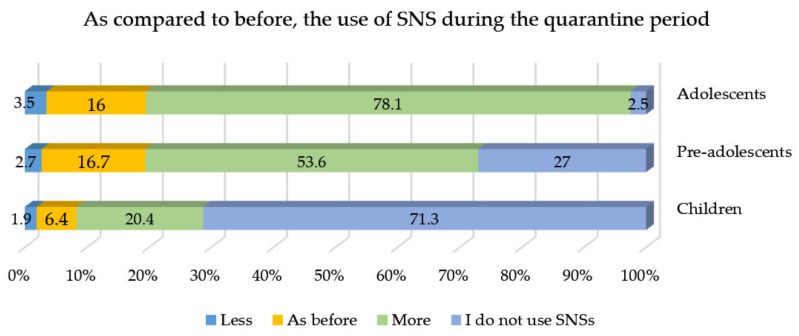
Frequency (%) of social network site (SNS) use during the quarantine period compared to before quarantine (*N* = 1047).

As shown in Figure 2, the frequency of SNS use was higher during the lockdown period, with adolescents as the most prolific users of SNSs, followed by pre-adolescents and children.

### 3.4. Compared to Before, How Do You Feel Now That You Are in Quarantine?

**Figure 3:**
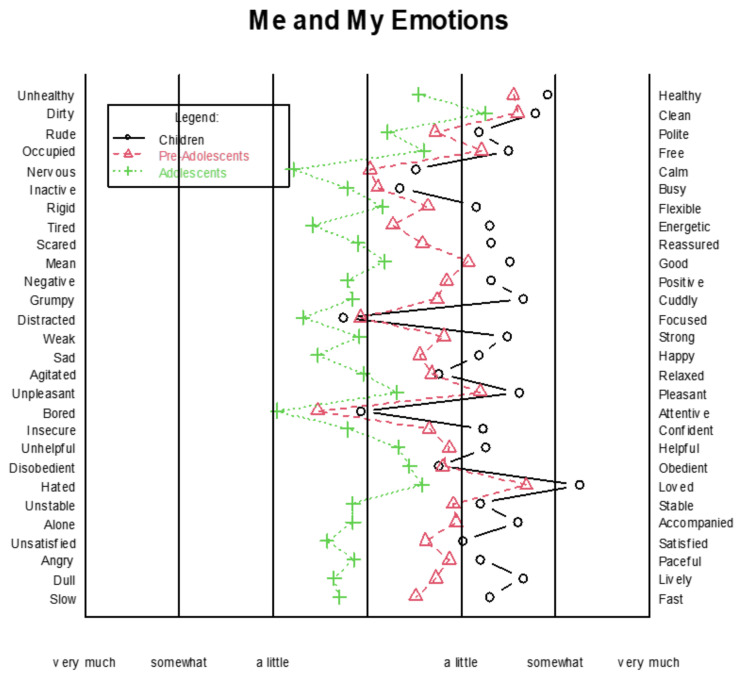
The trend of emotions reported by children (*n* = 265), pre-adolescents (*n* = 293), and adolescents (*n* = 489) during the quarantine period.

Figure 3, which was developed using R software, shows the trend of emotions reported by children, pre-adolescents, and adolescents during the quarantine period. Adolescents seem to have experienced more negative emotions compared to children and pre-adolescents. Specifically, children (*n* = 265) reported a mean of 5.02 (SD = 1.032) regarding the activity factor, a mean of 5.11 (SD = 1.24) for emotions, a mean of 5.37 (SD = 1.08) for the satisfaction factor, and a mean of 5.74 (SD = 1.00) for personality. Pre-adolescents (*n* = 293) presented a mean of 4.54 (SD = 1.03) for the activity factor, 4.64 (SD = 1.32) for emotions, 4.94 (SD = 1.07) for satisfaction, and 5.28 (SD = 1.05) for personality. Adolescents (*n* = 489) reported a mean of 3.82 (SD = 1.09) for activity, 3.69 (SD = 1.33) for the emotions factor, 4.22 (SD = 1.22) for satisfaction, and 4.40 (SD = 1.29) for the personality factor.

### 3.5. Kruskal–Wallis Test Assessing Age Differences

Table 1 displays the age differences (i.e., children, pre-adolescents, and adolescents) among the DS factors, for which the factors emotions, activity, personality, and satisfaction showed comparable trends. However, children experienced more positive emotions than pre-adolescents, who, in turn, experienced more positive emotions than adolescents. Moreover, adolescents reported a higher-than-usual use of SNSs than pre-adolescents and children, showing a particular preference for TikTok and Instagram. An opposite trend emerged as regards the habitual use of videogames; children reported using more videogames than pre-adolescents, who, in turn, used them more than adolescents.

### 3.6. Kruskal–Wallis Test Assessing Gender Differences

Table 2 shows gender differences among the DS factors. The emotions, activity, and personality factors were experienced more positively among males compared to females, whereas the satisfaction factor was experienced more positively by females. Moreover, females were more inclined to use SNSs, in particular TikTok and Instagram, compared to males. As regards the habitual use of videogames, males were more prone to use them compared to females.

## 4. Discussion

The aim of the present epidemiological study was to explore the perception of change and changing habits during the COVID-19 quarantine period among Italian children, pre-adolescents, and adolescents concerning several psychosocial dimensions, namely family life, important relationships, body functions, and school life. With this epidemiological study, we also focused on emotions and the use of SNSs, considering both gender and age differences.

Previous findings evidenced that the imposed home confinement was detrimental to children’s, pre-adolescents’, and adolescents’ physical health, leading to unhealthy sleep routines, sleep disturbances, and physical complaints [4,13]. In the current study, the results—assessed for the overall sample—showed that psychophysical aspects were not particularly affected by the emergency experience. The whole sample reported eating the same amount of food and not being hungrier than before; as far as sleeping and sleepiness are concerned, the sample percentages ranged from “less than before” to “as before”, thereby indexing a maintained and, for some, even an improved sleep routine. As previously observed [4,8,13], most of the participants missed practicing their favorite sports activities and reported exercising less, as expected under quarantine conditions. Nonetheless, the limited impact of the quarantine period on the investigated psychophysical aspects is somewhat unexpected, especially given that around 45% of the sample was from northern Italy, which was the most affected region by the virus, with the strictest and longest-lasting social restrictions. This further highlights the resilience of Italian youth, who proved to be adaptive and flexible during this critical period.

As regards school life, there was a general drop in motivation to study and do homework; most of the participants missed their school, teachers, and especially their classmates. Following their school routine at home was more complex due to the lack of a school context, which encourages children, pre-adolescents, and adolescents to stay focused and improves motivation. These findings are supported by previous studies that highlighted the presence of feelings of boredom, irritability, nervousness, restlessness. and difficulties in concentrating [4,5], all hindering youth motivation with respect to studying. In this regard, one can assume that the absence of in-person contacts with classmates and teachers could lead to a sense of uncertainty in students and teachers regarding school performance, thereby leading to feelings of anxiety and worry [8,26]. Moreover, all participants had to adapt to new and unusual ways of schooling, as lessons are traditionally held in a classroom, which allows youth to find a source of comradeship with their classmates, which was rendered difficult by the massive restrictions on in-person contacts. Indeed, relationships with peers are crucial for youth social development, as they offer an opportunity to discover the world outside the family life, which is especially important for the construction of adolescents’ self-identity [27]. In this context, digital devices played an important role in mediating peer relationships, as reported in a recent systematic review, suggesting that the use of such devices alleviates symptoms of loneliness and stress [28]. During the quarantine, the use of SNSs was higher among adolescents compared to children and pre-adolescents. More specifically, the use of SNSs, especially Instagram, as reported in previous studies [29], and TikTok, was more widespread among adolescents compared to children and pre-adolescents. However, as a result of the quarantine, children of all ages significantly increased their use of technology, although adolescents remained the most prolific users, followed by pre-adolescents and children. Moreover, females were more inclined to use SNSs, particularly TikTok and Instagram, compared to males, which is not surprising, considering that females use SNSs mainly for social purposes [30]. As regards the emotions experienced during the lockdown, a consistent trend emerged across the different age groups. Adolescents experienced more negative emotions compared to children and pre-adolescents. This negative trend might be due to a greater awareness of the emergency and of the uncertainty created by the virus, as well as the physiological uncertainty derived from the biopsychosocial changes experienced by adolescents [31]. This period is characterized by the development, processing, and regulation of more intense, complex, and variable feelings and emotions, representing fundamental growth goals [31] and comprising part of the process of identity formation. In this context, family, school, and peer groups represent different role models and sources of psychosocial conditioning [27,31], which, because of the pandemic, underwent a sudden change. The quarantine period represented a further challenge to this developmental period, requiring specific support to deal with its consequences. The overall findings of the present study suggest that although they exhibited a remarkable degree of flexibility and adaptability, it is necessary to preserve the emotional state and the relational well-being of children, pre-adolescents and, in particular, adolescents, taking into consideration their school and social life. The extent of the security measures, the uncertainty surrounding school life, and the massive social limitations imposed during the emergency period should encourage a debate on the need to develop ad hoc psychoeducational activities to support children, pre-adolescents, and adolescents both within the school context and outside. The timely implementation of such activities could help to mitigate the likely and, to an extent, still unseen long-term consequences resulting from the prolonged security measures and social restrictions.

## 5. Limitations and Strengths

The current study is not exempt from limitations. First, around 45% of the participants who answered the questionnaires come from northern Italy, whereas a smaller percentage were from southern and insular areas of Italy. Second, the sample is mainly comprised of females and adolescents. Moreover, a small percentage of parents completed the questionnaire on behalf of their children. In addition, we did not investigate whether all children in the sample had the same level of access to technological devices. Finally, although most of the sample live with their parents, it would be advisable to conduct an in-depth study among samples of children, pre-adolescents, and adolescents who lived in other environments (i.e., with grandparents, in rehab centers, etc.) during the lockdown period. Nevertheless, the present study is the first to explore and describe the changes in habits as perceived by Italian children, pre-adolescents, and adolescents, with a specific focus on emotions and SNS use, highlighting the importance of their perceptions during the quarantine period.

## 6. Conclusions

The overall findings suggest that although flexibility and adaptability were exhibited among the sample population, the emotional state and the relational well-being of children, pre-adolescents, and adolescents suffered as a result of pandemic restrictions. Owing to the current “developmental window” of the participants, the present findings should be considered with respect to implications for school life and all that it entails and represents for youth in terms of the environment in which they should develop and explore their relational skills and identity issues. Moreover, the increased use of technological devices and SNSs among children of all ages during the lockdown remains significant. Although such increased use was initiated and supported during the lockdown, it is important to keep in mind and prevent the risk that the use of these devices could become a substitute for both relational and in-person school contact. Overall, the obtained results underline the need to restore conditions allowing safe contacts with peers, in particular for adolescents, thereby encouraging a reflection on the effectiveness of distance learning during this developmental period. Future work is needed to investigate the effectiveness of distance learning among adolescents and to explore the impact of the ongoing changes in the school system. Considering the high use of SNSs during the period of isolation, especially among female adolescents, it is advisable to encourage them to find and practice alternative activities, which can direct them towards increased creativity. The long-term consequences of social restrictions and security measures, as well as the uncertainty surrounding school life, should be carefully considered and more broadly discussed to favor the development of ad hoc psychoeducational activities to support and further increase the resilience of children, pre-adolescents, and adolescents, who, despite the critical nature of the quarantine period, have nonetheless exhibited flexibility and adaptability.

## Figures and Tables

**Table 1:** Kruskal–Wallis test of age differences (*N* = 1047).

Age Differences			
	χ^2^	*p*-Value	Post Hoc Comparison
Emotions	190.12	<0.00	C > P > A
Activity	194.67	<0.00	C > P > A
Satisfaction	156.21	<0.00	C > P > A
Personality	210.06	<0.00	C > P > A
Habitual SNS use	497.85	<0.00	A > P > C
Since are you home for COVID-19, the time you spend on SNSs is…?	278.07	<0.00	A > P > C
TikTok	82.903	<0.00	A > P > C
Instagram	588.63	<0.00	A > P > C
Facebook	101.48	<0.00	ns
Twitter	49.44	ns	ns
Surfing the Internet, videos, tutorials	84.784	<0.00	A > P > C
Habitual videogames use	65.235	<0.00	C > P > A
Since are you home for COVID-19, the time you spend on the computer, TV, is…?	19.332	ns	ns

Note: ns = not significant, SNSs = social network sites, C = children, P = pre-adolescents, A = adolescents.

**Table 2:** Kruskal–Wallis test of gender differences (*N* = 1047).

Gender Differences			
	χ^2^	*p*-Value	Post Hoc Comparison
Emotions	23.448	<0.00	M > F
Activity	23.747	<0.00	M > F
Satisfaction	8.684	0.013	F > M
Personality	9.591	0.008	M > F
Habitual SNS use	42.985	<0.00	F > M
Since are you home for COVID-19, the time you spend on SNSs is…?	13.546	0.001	F > M
TikTok	78.939	<0.00	F > M
Instagram	33.03	<0.00	F > M
Facebook	1.798	ns	F = M
Twitter	0.185	ns	F = M
Surfing the Internet, videos, tutorial	1.1093	ns	F = M
Habitual videogames use	264.32	<0.00	M > F
Since are you home for COVID-19, the time you spend on the computer, TV, is…?	0.329	ns	F = M

Note: ns = not significant, SNSs = social network sites, F = females, M = males, *N* = 1047.

## Data Availability

The data presented in this study are available on request from the corresponding author. The data are not publicly available due to data privacy.
